# Developmental Context Determines Latency of MYC-Induced Tumorigenesis

**DOI:** 10.1371/journal.pbio.0020332

**Published:** 2004-09-28

**Authors:** Shelly Beer, Anders Zetterberg, Rebecca A Ihrie, Ryan A McTaggart, Qiwei Yang, Nicole Bradon, Constadina Arvanitis, Laura D Attardi, Sandy Feng, Boris Ruebner, Robert D Cardiff, Dean W Felsher

**Affiliations:** **1**Division of Oncology, Departments of Medicine and PathologyStanford University, Stanford, CaliforniaUnited States of America; **2**Division of Cellular and Molecular Tumor Cancer Center, Departments of Oncology and PathologyKarolinska Institute, StockholmSweden; **3**Department of Radiation and Cancer Oncology, Stanford UniversityStanford, CaliforniaUnited States of America; **4**Department of Surgery, Division of TransplantationUniversity of California, San Francisco, San Francisco, CaliforniaUnited States of America; **5**Department of Pathology, University of CaliforniaDavis, Davis, CaliforniaUnited States of America

## Abstract

One of the enigmas in tumor biology is that different types of cancers are prevalent in different age groups. One possible explanation is that the ability of a specific oncogene to cause tumorigenesis in a particular cell type depends on epigenetic parameters such as the developmental context. To address this hypothesis, we have used the tetracycline regulatory system to generate transgenic mice in which the expression of a *c-MYC* human transgene can be conditionally regulated in murine hepatocytes. *MYC*'s ability to induce tumorigenesis was dependent upon developmental context. In embryonic and neonatal mice, MYC overexpression in the liver induced marked cell proliferation and immediate onset of neoplasia. In contrast, in adult mice MYC overexpression induced cell growth and DNA replication without mitotic cell division, and mice succumbed to neoplasia only after a prolonged latency. In adult hepatocytes, *MYC* activation failed to induce cell division, which was at least in part mediated through the activation of p53. Surprisingly, apoptosis is not a barrier to *MYC* inducing tumorigenesis. The ability of oncogenes to induce tumorigenesis may be generally restrained by developmentally specific mechanisms. Adult somatic cells have evolved mechanisms to prevent individual oncogenes from initiating cellular growth, DNA replication, and mitotic cellular division alone, thereby preventing any single genetic event from inducing tumorigenesis.

## Abstract 2

A transgenic mouse model demonstrates that developmental context may be the reason why the spectrum of tumors differs in children and adults

## Introduction

The frequency of cancer development varies depending on the age of the host. In humans, the most common childhood cancers include tumors of the hematopoietic system, nervous system, and skeletal muscle system. In contrast, in the adult population, solid tumors of the lung, colon, breast, and prostate are more common. Differences in types of cancers in hosts of different ages may reflect the abundance of cells in a differentiative state susceptible to tumorigenesis (Greaves 1986; Klein and Klein 1986). Indeed, many reports document that oncogene activation generally induces tumorigenesis in immature cellular lineages (Adams et al. 1985; Spanopoulou et al. 1989; Pelengaris et al. 1999; Blyth et al. 2000).

We recently reported that upon oncogene inactivation tumor cells can differentiate into mature cells, and in this new differentiative context the reactivation of an oncogene fails to restore tumorigenesis (Jain et al. 2002). Based on these results, we speculate that only specific differentiative windows provide the correct epigenetic program to permit oncogene activation to initiate and sustain tumorigenesis. Here we directly evaluate whether an oncogene's ability to induce tumorigenesis depends on the differentiative context when this oncogene first becomes activated. We have examined the ability of the *C-MYC* oncogene to induce tumorigenesis in mice of different ages using a novel conditional transgenic model system for *C-MYC*–induced hepatocellular carcinoma (HCC).

*C-MYC* (now referred to as *MYC*) is a member of a family of proto-oncogenes comprising *C*-*MYC, N-MYC,* and *L-MYC. MYC* encodes a transcription factor that, as part of a heterodimeric complex with MAX, regulates the expression of a multitude of genes involved in regulating cellular proliferation and growth (Johnston et al. 1999; Grandori et al. 2000; Oster et al. 2002; Pelengaris et al. 2002a). Overexpression of MYC is commonly associated with tumorigenesis. MYC exerts its neoplastic function by inducing autonomous cellular proliferation and cellular growth, blocking differentiation, and inducing genomic destabilization (Dang 1999; Felsher and Bishop 1999a; Grandori et al. 2000; Oster et al. 2002; Pelengaris et al. 2002a; Karlsson et al. 2003). It is generally assumed that MYC is restrained from causing tumorigenesis because it concomitantly induces cellular proliferation and apoptosis (Pelengaris et al. 2002a).

HCC is a common and generally incurable human malignancy of epithelial cells (Thorgeirsson and Grisham 2002). HCC has been strongly associated with viral infections such as hepatitis B and C; exposure to toxins, such as alcohol, aflatoxin, and phenobarbital; and exposure to various carcinogens such as polyvinyl chloride (Thorgeirsson and Grisham 2002). Interestingly, the ability of these carcinogens, such as the hepatitis viruses, to induce HCC depends on the host age during infection. Hepatitis B infection acquired during neonatal development versus adulthood results in a several-magnitude increased risk of HCC (Chang et al. 1991). These results suggest that there are differentiative windows during liver development that may be more susceptible to neoplastic transformation.

Human tumors have been analyzed extensively for genetic events associated with HCC (Buetow et al. 1989; Tsuda et al. 1990; Boige et al. 1997; Piao et al. 1997). *MYC* oncogene activation is one of the more common events in the pathogenesis of HCC. MYC overexpression in human HCC is most commonly associated with genomic amplification (Abou-Elella et al. 1996; Kawate et al. 1999). Human HCCs exhibit amplification of *MYC* in up to 50% of tumors (Abou-Elella et al. 1996; Kawate et al. 1999). The presence of *MYC* amplification in HCC portends a more advanced and aggressive clinical phenotype (Abou-Elella et al. 1996). Thus, the *MYC* oncogene appears to play a critical role in the pathogenesis of HCC.

The most compelling evidence that *MYC* is causally associated with the etiology of HCC comes from animal models (Sandgren et al. 1989; Fourel et al. 1990; Murakami et al. 1993; Morgenbesser and DePinho 1994; Sargent et al. 1996, 1999; De Miglio et al. 1999; Santoni-Rugiu et al. 1999; Renard et al. 2000). *MYC* is frequently activated through insertional mutagenesis mediated by the hepadnavirus in woodchuck liver tumors (Fourel et al. 1990; Renard et al. 2000). Carcinogen-induced HCC in Wistar rats is associated with *MYC* amplification and overexpression (De Miglio et al. 1999). The overexpression of *MYC* (as a transgene) and other oncogenes (e.g., *RAS,* T antigen) in murine hepatocytes results in HCC (Sandgren et al. 1989). The latency of HCC in these transgenic mice is long, but is greatly accelerated by the transgenic overexpression of transforming growth factor-alpha (Murakami et al. 1993; Sargent et al. 1996, 1999; Santoni-Rugiu et al. 1999). These results highlight that the activation of *MYC* alone is not sufficient to induce HCC.

Traditional transgenic systems that have been previously used to study the role of oncogenes in tumorigenesis continuously overexpress transgenes and hence preclude the investigation of the initial and developmentally specific consequences of oncogene activation. To investigate the developmentally specific consequences of MYC overexpression in the pathogenesis of HCC in vivo, we used transgenic mice in which the MYC proto-oncogene is conditionally regulated via the tetracycline regulatory system (Tet system) (Felsher and Bishop 1999b). We found that the ability of *MYC* to induce cellular proliferation versus cellular growth, and consequently its ability to induce tumorigenesis in murine hepatocytes, was dependent on the age of the host. Our results have implications for the mechanisms by which *MYC* and other oncogenes initiate and are restrained from causing tumorigenesis.

## Results

### A Conditional Model System for *MYC*-Induced HCC

**Figure 1:**
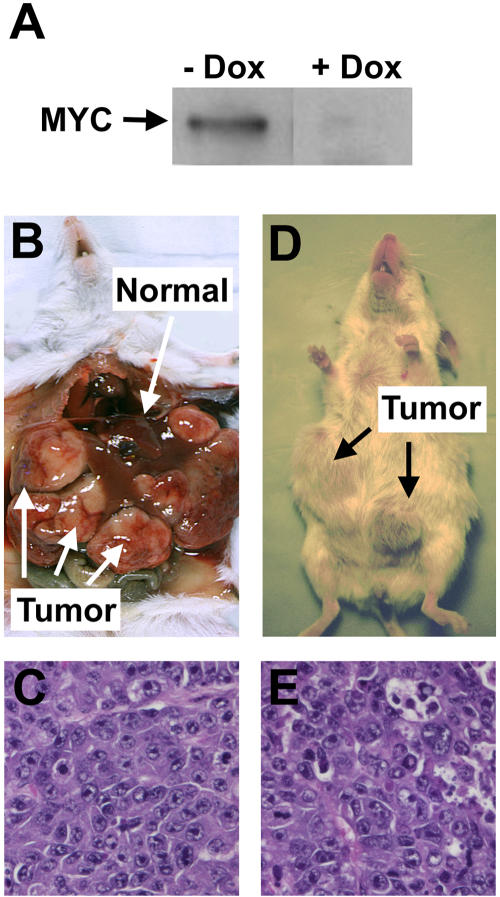
MYC Overexpression in Adult Hepatocytes Results in HCC (A) Western blot analysis demonstrating that mice transgenic for both LAP-*tTA* and TRE-MYC conditionally express *MYC* protein in their hepatocytes in the absence (−) but not in the presence (+) of doxycycline. (B) Adult mouse with *MYC*-induced liver tumor. (C) Histology of an adult *MYC*-induced liver tumor. (D) Gross pathology of an adult liver tumor transplanted subcutaneously into a *scid* mouse. (E) Histology of an adult tumor transplanted subcutaneously into a *scid* mouse.

We used the Tet system to conditionally express *MYC* in murine hepatocytes (Kistner et al. 1996; Felsher and Bishop 1999b). We mated the transgenic line TRE-*MYC* (Felsher and Bishop 1999b), which contains the tetracycline response element adjacent to the human *MYC* cDNA, with the transgenic line LAP-*tTA* (Kistner et al. 1996), which contains a liver-specific enhancer that drives the expression of the tetracycline-transactivating protein. Mice possessing both transgenes exhibited increased expression of the *MYC* transgene in their hepatocytes (Figure 1A). Mice possessing either transgene alone did not overexpress *MYC* and lacked evidence of morbidity or mortality. Similarly, mice possessing both transgenes that were treated with doxycycline to suppress *MYC* transgene expression did not exhibit a phenotype. Thus, we have developed a transgenic model that enables us to conditionally regulate *MYC* expression in murine hepatocytes.

**Figure 2:**
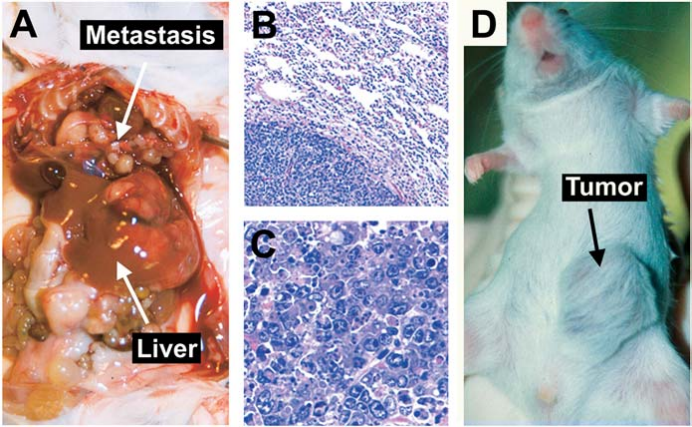
MYC-Induced Hepatic Tumors Are Invasive and Metastatic (A) Adult mouse with *MYC*-induced liver tumor that has metastasized to the abdomen and the lungs. (B) Histology of an adult *MYC*-induced lung metastasis. (C) Histology of an adult *MYC*-induced liver tumor. (D) Gross pathology of a liver tumor from a neonatal host transplanted subcutaneously into a *scid* mouse.

To investigate if MYC overexpression is sufficient to induce HCC in our model system, we removed doxycycline treatment in adult mice (6–12 weeks of age) transgenic for both TRE-*MYC* and LAP-*tTA*. Ninety percent of adult mice overexpressing MYC succumbed to liver tumors with a mean latency of 35 weeks. At necropsy, mice exhibited marked gross enlargement of the liver with multiple tumor masses (Figure 1B). The normal liver architecture was disrupted by nodular tumors with histological features typical of HCC. Tumors were composed of dysplastic nests of cells with large pleomorphic nuclei, delicate vesicular chromatin, and very prominent nucleoli (Figure 1C). Tumors could invade into the abdomen and the lung (Figure 2A–2C). These features demonstrated that MYC overexpression in adult mice resulted in HCC.

To confirm that these tumors were malignant, we transplanted them subcutaneously into *scid* mice. Tumors formed in the inoculated mice after an 8–10-week latency (see Figure 1D). The transplanted tumors displayed identical histology to the primary transgenic tumor (see Figure 1E versus 1C). Normal adult hepatocytes failed to induce tumors when inoculated into *scid* mice. We conclude that MYC overexpression in adult hepatocytes results in the formation of highly malignant liver cancers with features consistent with human HCC.

### Developmental State of the Host Influences on Frequency and Latency of Tumor Onset

**Figure 3:**
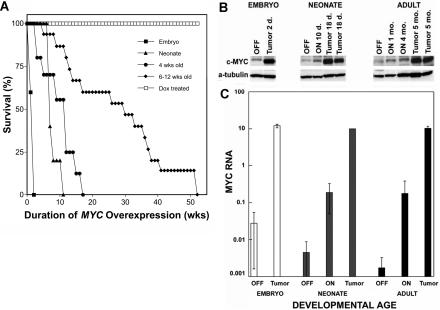
MYC's Ability to Induce HCC Is Inversely Correlated with the Age of the Host at the Time of *MYC* Activation (A) Survival of transgenic mice demonstrates that tumorigenesis in the liver is inversely correlated with the age of the host at the time of *MYC* induction. Shown are cases where *MYC* was constitutively expressed (▪), newborn mice in which *MYC* was activated at birth (▴), young mice in which *MYC* was activated at 4 weeks of age (•), adult mice in which *MYC* was activated at 6–12 weeks of age (♦), and transgenic mice treated with doxycycline (□). Cohorts consisted of 15–30 mice. Mice were scored when moribund. *MYC* transgene expression was induced to similar levels in the differently aged cohorts of mice. Survival time is measured as the time after *MYC* induction. (B) Western blot examining total MYC protein levels (human MYC and endogenous murine c-MYC) in mice when *MYC* is induced during embryonic development, activated at birth, and activated during adulthood (after 10 weeks of age). Adult mice exhibited a progressive increase in MYC protein levels during the course of *MYC* induction, with a significant increase in MYC protein in tumors. MYC protein levels in neonatal mice in which *MYC* was activated at birth were slightly increased at 10 d of age, and significantly increased at 18 d of age when these mice developed liver tumors. Liver tumors in 2- and 6-d-old neonatal mice that overexpressed MYC during embryonic development exhibited MYC protein levels similar to those observed in neonatal and adult tumors. (C) Real-time PCR analysis showing human *MYC* RNA levels in mice after different durations of *MYC* transgene induction. Adult livers exhibited a small increase in *MYC* RNA levels upon *MYC* activation, and a much greater increase in *MYC* RNA in MYC-induced tumors (black bars). In neonatal mice in which the *MYC* transgene was induced at birth, *MYC* RNA levels rose after 10 d of *MYC* activation. When these neonatal mice developed liver tumors, they exhibited *MYC* RNA levels similar to those seen in adult tumors (gray bars). Mice in which *MYC* was overexpressed during embryonic development developed liver tumors by 2 d of age and exhibited *MYC* RNA levels similar to those observed in neonatal and adult tumors (white bars).

To determine if the developmental state of host hepatocytes influenced the ability of *MYC* activation to induce tumorigenesis, we induced *MYC* in cohorts of different ages (Figure 3A). Mice that overexpressed MYC during embryonic development of the liver succumbed to neoplasia within 10 d of birth. Mice in which *MYC* was activated at birth (neonates) succumbed to neoplasia within 8 weeks. Mice in which *MYC* was induced at 4 weeks or 6–12 weeks of age developed tumors after a mean latency of 15 and 35 weeks, respectively (Figure 3A). We conclude that the ability of *MYC* activation to induce tumorigenesis in hepatocytes is inversely correlated with the developmental age of the host.

One possible explanation for our results was that the levels of MYC induction were different in embryonic and neonatal versus adult hosts. To address this possibility, we examined total MYC protein levels by Western analysis using a polyclonal antibody that recognizes both the human c-MYC protein and the endogenous murine c-MYC (Figure 3B). In neonatal and adult mice, MYC protein levels were induced at similar levels (Figure 3B). In tumors from embryos, neonatal, and adult mice, MYC protein levels increased an additional 5- to 10-fold over the levels observed in nontransgenic and in *MYC*-induced nonneoplastic livers. Tumorigenic conversion of hepatocytes was associated, in all age groups of mice, with further increases in the levels of MYC protein (Figure 3B). We obtained similar results by quantitative PCR analysis of mRNA expression of the human *MYC* transgene (Figure 3C). In neonatal and adult mice, *MYC* transgene expression was induced at similar levels. In tumors from embryos, neonatal, and adult mice, the levels of *MYC* transgene RNA increased an additional 10-fold over the levels observed in nonmalignant hepatocytes (Figure 3C). Hence, tumorigenic conversion of hepatocytes was associated, in all age groups of mice, with further increases in the levels of *MYC* transgene expression. However, differences in the ability of *MYC* activation to initiate tumorigenesis in mice of different ages did not appear to be related to differences in the levels of induction of *MYC* transgene expression. The increased levels of MYC expression we observed in tumors likely reflect that the proliferating tumor cells express more abundant levels of the *MYC* transgene than normal hepatocytes. This observation is consistent with observations described in other transgenic models in which expression of transgenes is generally higher in tumors than it is in the normal cellular counterparts (Weiss et al. 2003).

### *MYC* Activation in Embryonic and Neonatal Hepatocytes Induces Cellular Proliferation and Tumorigenesis

**Figure 4:**
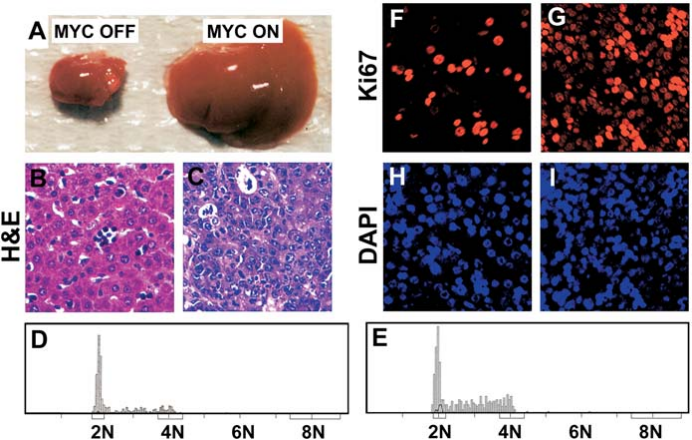
*MYC* Activation during Embryonic Development Induces a Rapid Onset of Neoplasia (A) A normal neonatal liver and a neonatal liver in which *MYC* was activated embryonically. (B) Histology of a normal neonatal liver in which *MYC* was not activated. (C) Histology of a neonatal liver in which *MYC* was activated embryonically. (D) DNA content of normal neonatal hepatocytes. (E) DNA content of neonatal hepatocytes in which *MYC* was activated embryonically. (F and H) Ki67 immunofluorescence and DAPI staining corresponding to a normal neonatal liver. (G and I) Ki67 immunofluorescence and DAPI staining corresponding to a neonatal liver in which *MYC* was activated embryonically.

**Figure 8:**
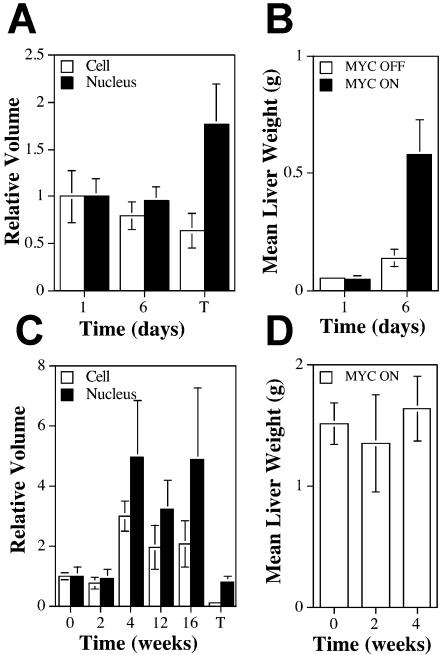
*MYC* Activation in Adult Hepatocytes Causes Cellular Hypertrophy (A) Relative volumes of neonatal hepatocytes and nuclei after *MYC* activation. Data are expressed as normalized volume plus or minus the standard error of the mean. The volume was normalized by dividing each measurement by the mean volume of normal 1-d-old neonatal mice. Three livers were measured per time point. T, tumor. (B) Neonatal liver weights of normal and *MY*C-activated livers. Three to five livers were weighed per time point. Data are expressed as the mean weight (grams) plus or minus the standard error of the mean. (C) Relative volumes of adult hepatocytes and nuclei after *MYC* activation. Volumes of cells are expressed as the mean volume divided by the mean volume of hepatocytes from normal mice plus or minus the standard error of the mean. Cells from two to three livers were measured per time point. (D) Adult liver weights after *MYC* activation. A total of nine livers were measured per time point after *MYC* activation. Data are expressed as the mean weight (grams) plus or minus the standard error of the mean.

To evaluate how *MYC*'s ability to induce tumorigenesis is influenced by the age of mice, we investigated the initial consequences of *MYC* activation in hepatocytes during different developmental periods. Mice that overexpressed MYC during embryonic development were born with livers similar in weight and gross architecture to normal age-matched livers, yet exhibited increasing numbers of neoplastic cells from birth through the first week of life associated with progressive abdominal enlargement. At necropsy, abdominal enlargement was associated with marked hepatomegaly with a 5-fold increase in total liver weight (Figure 4A, and see Figure 8B below). Although these livers were larger, the gross architecture was preserved (Figure 4A, MYC ON versus MYC OFF). When we examined the histology of the livers in which MYC was overexpressed during embryogenesis, we found that they resembled liver cancers (Figure 4B versus 4C) similar to the *MYC*-induced HCCs we observed in adult mice (see Figure 1C versus 4C). Hence, MYC overexpression appears to induce cellular proliferation in neonatal hepatocytes that progresses rapidly to neoplasia.

To determine if MYC was inducing changes in cell cycle transit, we measured the DNA content of isolated nuclei from normal hepatocytes and hepatocytes in which *MYC* was activated during embryonic development (Figure 4D and 4E). Normal neonatal hepatocytes mostly contained 2N DNA content, consistent with most of the cells residing in G1 (Figure 4D). A minority of hepatocytes exhibited 4N and 2N–4N DNA content, demonstrating that few cells were in G2/M and S phase, respectively. In contrast, upon *MYC* activation the proportion of neonatal hepatocytes with 2N–4N DNA content substantially increased, suggesting that an increased number of cells were in S phase (Figure 4E).

In order to confirm that *MYC* activation caused tumorigenesis by inducing cell proliferation, we performed Ki67 immunofluorescence and DAPI staining in tumors induced by *MYC* activation during embryonic development and in age-matched nontransgenic livers. Indeed, there was evidence for increased hepatocyte proliferation in the MYC-induced neonatal tumor, as demonstrated by an increase in Ki67-positive cells (Figure 4G and 4I versus 4F and 4H). We conclude that *MYC* activation during embryonic development causes neonatal hepatocytes to undergo DNA replication, cell cycle transit, proliferation, and almost immediate neoplastic conversion.

To confirm that *MYC* activation during embryonic development induced tumorigenesis in neonatal livers, we transplanted neoplastic hepatocytes into *scid* mice. We found that neoplastic neonatal hepatocytes readily formed tumors, whereas the transplantation of normal neonatal hepatocytes did not form tumors (see Figure 2D and unpublished data). Therefore, *MYC* overexpression during embryonic development of the murine liver causes hepatocellular tumorigenesis within the first 10 d of birth (Figure 3). We conclude that *MYC* overexpression results in rapid neoplastic conversion of neonatal hepatocytes.

**Figure 5:**
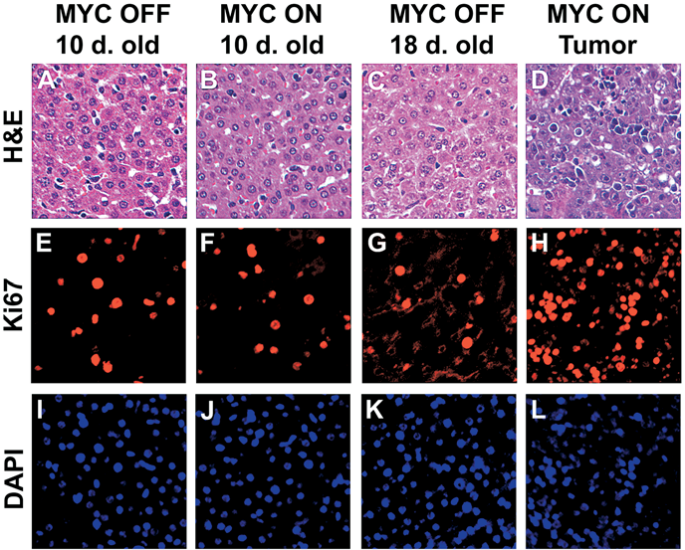
*MYC* Activation at Birth Induces Proliferation of Neonatal Hepatocytes (A) Histology of a normal 10-d-old neonatal liver. (B) Histology of a 10-d-old liver in which *MYC* was activated at birth. (C) Histology of a normal 18-d-old neonatal liver. (D) Histology of a MYC-induced neonatal liver tumor that developed after 18 d of MYC overexpression; *MYC* was activated at birth. (E–L) Ki67 immunofluorescence (E–H) and DAPI staining (I–L) of normal neonatal hepatocytes (E, G, I, and K), *MYC*-activated hepatocytes (F and J), and MYC-induced neonatal tumors (H and L). Upon initial *MYC* activation in neonatal mice, there was a small increase in Ki67-positive cells. MYC-induced neonatal tumors exhibited much higher levels of Ki67-positive cells.

Mice in which *MYC* was activated at birth exhibited progressive abdominal enlargement during their second and third weeks of life, and they showed signs of tumorigenesis by 18 to 40 d of age. When these mice developed tumors their livers were ten times the normal size, were paler, exhibited a multitude of coalescing tumor nodules, and preserved a normal gross architecture (unpublished data). We did not observe any histological changes in the liver after 10 d of *MYC* activation (Figure 5A versus 5B); however, by 18 d of *MYC* activation the histology resembled liver cancers (Figure 5C versus 5D), similar to the MYC-induced HCCs we observed in adults (Figure 5D versus 1C).

We performed Ki67 immunofluorescence and DAPI staining in order to determine if MYC overexpression in neonatal livers was inducing hepatocyte proliferation. At 10 d of age the liver is in an active state of proliferation; thus, there was little detectable difference in the number of Ki67-positive cells between the *MYC*-activated and nontransgenic livers (Figure 5E and 5I versus 5F and 5J). However, once these livers became neoplastic, there was a great increase in the number of Ki67-positive cells (Figure 5G and 5K versus 5H and 5L).

### *MYC* Activation in Adult Hepatocytes Induces Cellular Growth, but Not Proliferation

**Figure 6:**
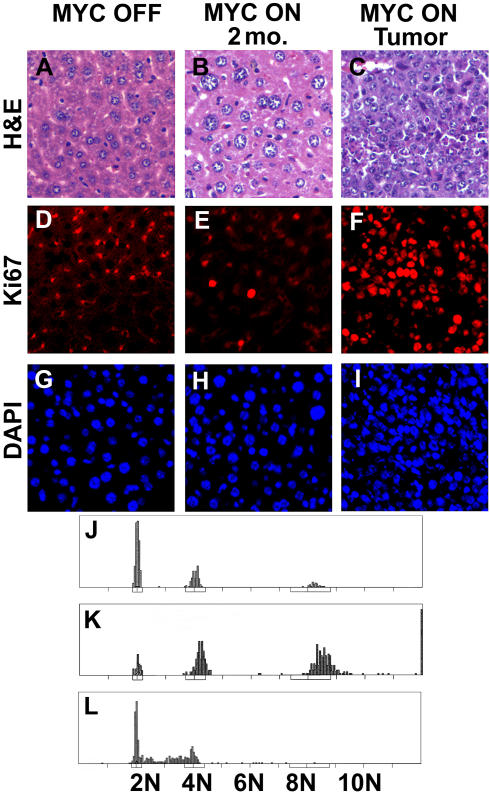
*MYC* Activation in Adult Hepatocytes Induces Increased Cell Size and Endoreduplication, and Only Results in Cell Proliferation upon Neoplastic Conversion of Hepatocytes (A) Histology of a normal liver. (B) Histology of a liver 2 months after *MYC* activation. (C) Histology of a MYC-induced liver tumor. (D and G) Ki67 immunofluorescence and DAPI staining of a normal adult liver. (E and H) Ki67 and DAPI staining of an adult liver after 8 weeks of *MYC* activation. (F and I) Ki67 and DAPI staining of a MYC-induced adult tumor. (J) DNA content measured in normal hepatocytes. (K) DNA content measured after *MYC* induction for 2 months. (L) DNA content of a representative MYC-induced liver tumor.

**Figure 7:**
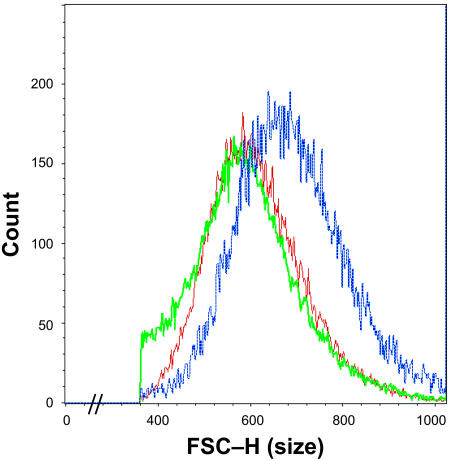
*MYC* Activation in Adult Hepatocytes Induces Increased Cell Size A histogram obtained by FACS forward versus light scatter analysis of adult hepatocytes from normal FVB/N livers (green), doxycycline-treated transgenic livers (red), and livers in which the *MYC* transgene was overexpressed for 3 months (blue). The *x*-axis represents cell size and the *y*-axis represents cell count. Adult mice were matched for age.

We examined the initial consequences of MYC overexpression in adult hepatocytes. In contrast to the rapid neoplastic conversion we observed in embryonic or neonatal hepatocytes (Figures 4 and 5), MYC overexpression in adult hepatocytes caused a marked cellular growth, accompanied by an even greater relative nuclear growth, as observed by histological analysis (Figures 6–8). The effects of MYC overexpression on the size of adult hepatocytes depended on the duration of *MYC* activation. After 2 weeks of MYC overexpression, no changes were observed in cell size compared to normal hepatocytes (Figure 8C and unpublished data). However, after 4–8 weeks of *MYC* activation, adult hepatocytes exhibited increased cell and nuclear size (Figure 6B versus 6A and Figure 8C). Similar results were observed in over 20 different mice. Similarly, we observed that MYC induces hypertrophy of hepatocytes by flow cytometry analysis (Figure 7). Further duration of *MYC* activation did not induce further cell growth, as measured up to 50 weeks of *MYC* activation (unpublished data). Thus, there may be an absolute limit to the ability of *MYC* to induce liver growth. *MYC* activation in adult hepatocytes was not associated with a change in overall liver weight (Figure 8D). Since the cells were bigger, but the overall weight of the liver did not increase, we infer that the total number of hepatocytes was unchanged or slightly decreased. One possible explanation for these results is that MYC induced apoptosis, as described below.

To examine if *MYC* activation induced proliferation of adult hepatocytes, we measured Ki67 expression by immunofluorescence. We did not observe increased Ki67 expression when MYC was overexpressed in the adult liver (Figure 6E and 6H versus 6D and 6G). Only upon neoplastic conversion of hepatocytes was there evidence for increased hepatocyte proliferation (Figure 6F and 6I). We conclude that MYC overexpression in adult hepatocytes induces increased nuclear and cell growth, but not cell proliferation. Our observations are consistent with previous reports that *MYC* activation induces cell growth (Mateyak et al. 1997; Iritani and Eisenman 1999; Johnston et al. 1999; Grandori et al. 2000; Kim et al. 2000).

*MYC* activation in adult hepatocytes eventually culminated in tumorigenesis, demonstrating that some adult hepatocytes acquire the ability to undergo cell division. To confirm this, we measured the nuclear and cellular sizes in liver tumors. When we examined the cell size in ten different tumors from adult hosts, we found that in all tumors, the cell size was reduced to below normal and the nuclear size was similar to that of normal hepatocytes (Figures 6C and 8C). We were also able to confirm that the cell size of tumor cells was reduced to below normal by FACS forward versus side scatter (unpublished data). We conclude that *MYC*-induced malignant conversion of adult hepatocytes is associated with the acquired ability to undergo mitotic division.

### MYC Overexpression in Adult Hepatocytes Results in Endoreduplication

To further define the consequences of *MYC* activation on the cell cycle, we examined the DNA content of isolated nuclei from normal and *MYC*-activated adult hepatocytes. We expected that if adult hepatocytes were restrained from undergoing mitotic division, *MYC* activation might result in endoreduplication. Age-matched normal hepatocytes exhibited a 2N DNA content consistent with most of the cells residing in G1, and there was no evidence for cells in S or G2/M (Figure 6J). After *MYC* activation for 2 months, we found that almost all nuclei had a 4N, 8N, or 12N DNA content, suggesting that the cells replicated their DNA repeatedly without dividing (Figure 6K). Almost no cells contained the intermediate DNA content (2N–4N), demonstrating that very few cells were in S phase at any given time. We conclude that *MYC* activation induces endoreduplication of the genomes of normal adult hepatocytes. Our results are consistent with reports that MYC overexpression can enforce DNA replication, resulting in endoreduplication in normal cells (Cerni et al. 1986; Mai et al. 1996; Chernova et al. 1998; Felsher et al. 2000).

We reasoned that if *MYC* was causing endoreduplication in adult hepatocytes by arresting cell division and enforcing DNA replication, then upon neoplastic conversion these hepatocytes must acquire the ability to undergo mitotic division and would no longer endoreduplicate. As predicted, tumors did not exhibit evidence for endoreduplication (Figure 6L). Greater than 70% of the tumor cells contained a 2N–4N DNA content and none of the cells contained greater than 4N DNA content. The majority of tumor cells were in S phase (Figure 6L). Hence, *MYC*-induced tumorigenesis in adult hepatocytes is associated with the acquired ability to divide mitotically.

### MYC Overexpression in Adult Hepatocytes Does Not Induce Apoptosis

**Figure 9:**
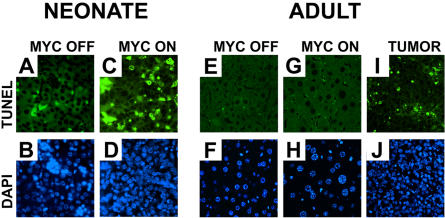
*MYC* Activation Does Not Induce Apoptosis in Murine Hepatocytes TUNEL assay (A, C, E, G, and I) and DAPI staining (B, D, F, H, and J) of normal (A, B, E, and F) and *MYC*-activated (C, D, and G–J) hepatocytes of neonatal (A–D) and adult (E–J) mice. After 4 weeks of *MYC* activation in adult hepatocytes, there was no evidence of apoptosis either by TUNEL assay (G) or by DAPI staining of nuclei (H). *MYC* activation is associated with increased apoptosis with the neoplastic conversion of neonatal (C) and adult (I) hepatocytes. Representative data from one of three experiments are shown. Identical results were seen when *MYC* was activated for 2 or 8 weeks.

*MYC*-induced apoptosis is an important mechanism that restrains *MYC* from causing tumorigenesis (Evan et al. 1992; Pelengaris et al. 2000, 2002b). We reasoned that MYC may induce cellular hypertrophy, but not an increase in liver mass, because MYC induces apoptosis. Normal neonatal and adult hepatocytes did not undergo apoptosis, as demonstrated by TUNEL assay or DAPI staining (Figure 9A, 9B, 9E, and 9F). Surprisingly, we could not find evidence that *MYC* induced apoptosis in adult hepatocytes by TUNEL assay or DAPI staining after 2, 4, or 8 weeks of *MYC* induction prior to tumor formation (Figure 9G and 9H and unpublished data). Contrary to what we expected, *MYC* activation was only associated with increased apoptosis in the hepatocytes of liver cancers (Figure 9C, 9D, 9I, and 9J). Hence, apoptosis is not necessarily the mechanism restraining *MYC* from causing tumorigenesis, at least in hepatocytes (Pelengaris et al. 2002a). However, we recognize that MYC could be inducing low levels of apoptosis in hepatocytes, perhaps not easily detected by TUNEL, since apoptotic cells may be rapidly eliminated from the liver through the host reticulo-endothelial system. Such a low level of apoptosis still could explain why in the adult liver hepatocytes become hypertrophic but the liver mass does not increase.

### Loss of p53 Function Cooperates with *MYC* to Induce Tumorigenesis in Adult Mice

**Figure 10:**
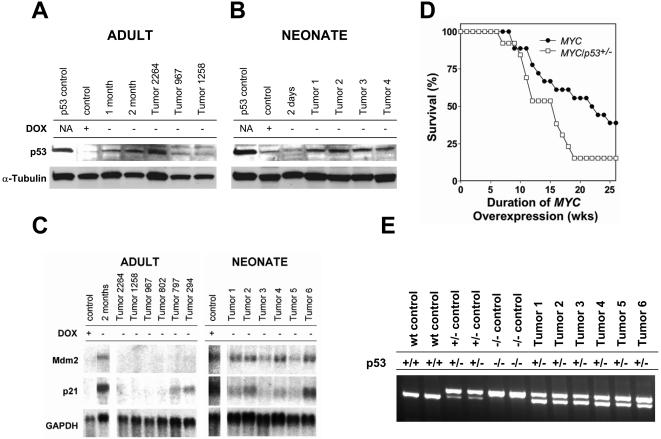
*MYC* Activation Induces p53 Function, and Loss of p53 Function Is Necessary for MYC to Induce Tumorigenesis in Adult Hepatocytes (A) Western blot analysis for p53 protein expression after *MYC* activation for 1 month, 2 months, and in MYC-induced tumors. As a positive control, we used a lymphoma cell line that overexpresses a mutant p53, kindly provided by Dr. Kevin Smith. (B) Western blot analysis for p53 protein expression in a normal neonatal liver, in a neonatal liver in which MYC was activated during embryonic development that was obtained from a 2-d-old mouse, and in MYC-induced neonatal tumors. As a positive control, we used a lymphoma cell line generated in our lab that overexpresses a mutant p53. (C) Northern blot analysis for *p21* and *MDM2* in neonatal and adult liver tumors. (D) Survival of adult mice after activation of *MYC* in the presence of the wild type or the loss of one *p53* allele. (E) Loss of heterozygosity analysis of *MYC/p53^+/−^* tumors by PCR analysis.

Previously, we have shown that the loss of p53 function is required to permit the cell division of normal mouse and human fibroblasts overexpressing MYC (Felsher et al. 2000). We speculated that loss of p53 function might be similarly required for *MYC* activation to induce cell proliferation and tumorigenesis in hepatocytes. First, we examined if *MYC* activation affected p53 protein expression. We found that *MYC* activation was associated with an increase in p53 protein levels in adult hepatocytes, as measured by Western analysis (Figure 10A). Conversely, tumors in adult mice frequently exhibited reduced levels of p53 protein expression (Figure 10A and unpublished data). Not all tumors exhibited reduced p53 expression. Since p53 mediates its function largely through inducing the transcription of many different genes, we evaluated if tumors exhibited a loss of p53 function by measuring the expression of these target genes. We found by Northern analysis that the p53 target genes, *p21* and *MDM2,* were induced upon *MYC* activation in adult livers (Figure 10C). Conversely, *MYC*-induced adult HCCs frequently exhibited reduced or no expression of p53 downstream targets. Notably, a tumor that exhibited high p53 protein levels, tumor 2264, exhibited a loss in expression of p53 target genes. In contrast, tumors arising in neonatal mice expressed p53 protein and exhibited the induction of p53 target genes (Figure 10B and 10C). We conclude that the adult, but not neonatal *MYC*-induced liver tumors require the loss of p53 function for tumorigenesis. Hence, HCCs that arise in adult versus neonatal hosts appear to occur through genetically distinct mechanisms.

To directly address if loss of any of p53's functions accelerates the ability of *MYC* to induce HCC in adult mice, we generated transgenic mice that overexpressed *MYC* in their hepatocytes in the absence of one *p53* allele. We mated LAP-*tTA*/TRE-*MYC* mice with *p53^+/−^* mice that were in the FVB/N background. We activated *MYC* in mice when they were 6 weeks old and monitored them for morbidity. We found that even the loss of a single *p53* allele was sufficient to reduce the mean latency of tumor onset in 6-week-old adult mice from 20 weeks to 15 weeks (Figure 10D). We found by PCR that tumors did not generally inactivate the second allele through deletion (Figure 10E). Our results extend previous findings that suggest that the lack of p53 function cooperates with MYC to induce HCC (Klocke et al. 2001). We conclude that even a slight reduction of p53 function greatly facilitates the ability of MYC to induce tumorigenesis in adult hepatocytes.

### Partial Hepatectomy Accelerates MYC-Induced Tumorigenesis

**Figure 11:**
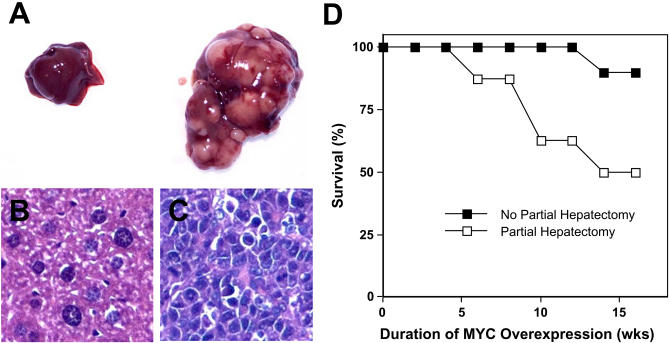
Partial Hepatectomy Accelerates the Ability of MYC to Induce HCC in Adult Mice (A) Liver from an adult mouse 7 weeks after *MYC* activation exhibited no gross phenotypic changes (left), whereas the liver from an adult mouse 7 weeks after *MYC* activation that had undergone partial hepatectomy exhibited a multifocal liver tumor (right). (B) The histology of the liver from an adult mouse 7 weeks after *MYC* activation exhibited no evidence of a tumor. (C) The histology of a liver from an adult mouse after *MYC* activation that had undergone partial hepatectomy exhibited a multifocal HCC. (D) Survival after *MYC* activation in adult mice that have either not undergone surgery (▪) or undergone a partial hepatectomy (□). Results are pooled from two independent experiments with a total of ten mice per group.

Our results suggest that the ability of MYC to induce tumorigenesis in hepatocytes depends on the developmental context. We recognized that an alternative explanation is that MYC induces tumorigenesis more readily in hepatocytes that are already proliferating. Adult hepatocytes are known to undergo rapid proliferation in response to partial hepatectomy resulting in the complete regeneration of the liver within 2 weeks of surgical removal (Michalopoulos and DeFrances 1997; Kountouras et al. 2001). We found that *MYC* activation in adult mice that have undergone partial hepatectomy exhibited a reduced latency of tumor induction in comparison with adult mice that had not undergone surgery (mean latency of 14 weeks versus 35 weeks). However, this latency of tumorigenesis in adult mice after partial hepatectomy was still up to two magnitudes longer than what was observed when *MYC* was activated in embryonic and neonatal mice (<10 d and 4 weeks, respectively) (Figures 11 and 3A). In addition, tumors in mice that had undergone partial hepatectomy, unlike tumors arising in neonatal mice, were multifocal, suggesting that tumorigenesis was occurring infrequently (Figure 11A versus 4A). Similarly, upon histological analysis, after *MYC* was activated for 7 weeks in mice that had undergone partial hepatectomy, mice exhibited many individual foci of HCC (Figure 11B and 11C). Finally, in adult mice after partial hepatectomy, but not in neonatal mice, areas of the liver that had not undergone neoplastic conversion clearly exhibited increased cellular hypertrophy, and hence were unable to undergo mitotic division (unpublished data). We conclude that the ability of MYC to induce tumorigenesis in adult hepatocytes is accelerated after partial hepatectomy when adult hepatocytes are proliferating, but other developmentally specific parameters are more important in determining when oncogene activation will induce tumorigenesis than the ability of hepatocytes simply to proliferate.

## Discussion

### Developmental Context Influences *MYC*'s Ability to Induce Cellular Proliferation

Here we demonstrate that *MYC*'s ability to induce cellular growth versus proliferation and tumorigenesis depends on the differentiative context of a cellular lineage. Our results have general implications for the mechanisms by which *MYC* and other oncogenes induce and are restrained from causing tumorigenesis.

We found that the initial consequences of MYC overexpression, as well as its ability to induce tumorigenesis in murine hepatocytes, depend on the host age. The activation of *MYC* during embryonic development and at birth caused gross liver enlargement and rapid emergence of neoplasia. This increased liver size resulted from increased cell number or cellular hyperplasia. In contrast, in adult murine hepatocytes, *MYC* activation induced cell growth, resulting in cellular hypertrophy and endoreduplication. MYC overexpression caused HCC in adult mice less frequently than in neonates, and only after a prolonged latency. We conclude that the ability of *MYC* to induce cellular proliferation and tumorigenesis appears to be determined by the relative developmental maturation of a cellular lineage.

We recognized that an alternative explanation for our results is that only hepatocytes that are actively proliferating are susceptible to neoplastic transformation upon *MYC* induction. We were able to address this possibility directly by examining the consequences of MYC overexpression in adult hepatocytes after partial hepatectomy, which resulted in the immediate induction of adult hepatocyte proliferation associated with complete liver regeneration within 2 weeks. Adult mice only exhibited a modest increased susceptibility to *MYC*-induced tumorigenesis observed when *MYC* was induced in embryonic mice. This is despite the fact that after partial hepatectomy the total number of proliferating hepatocytes would be at least two magnitudes greater than in a neonatal or embryonic liver, whose total mass is much smaller. We conclude that developmentally specific parameters other than proliferation are more likely to play a causative role in the differential susceptibility of embryonic and neonatal hepatocytes to *MYC*-induced tumorigenesis.

Several different development-related factors could account for our observations. The relative ability of MYC to induce tumorigenesis in developmentally immature hosts may reflect the relative increased abundance of immature hepatocytes. Homeostatic mechanisms that regulate liver size and hepatocyte proliferation may permit MYC-induced proliferative expansion in the developing livers of young hosts and restrain proliferation in the mature liver of adult hosts. Differences in rates of tumorigenesis may be related to telomerase function that monitors the distance from senescence, as recently described (Artandi et al. 2000). An unlikely possibility is that higher doses of tetracyclines may impede liver carcinogenesis (NTP 1989). The conditional model system that we have described should be useful for addressing these different possible mechanisms.

We conclude that mechanisms that regulate mitotic division play a critical role in preventing potent oncogenes, such as *MYC,* from inducing cancer in adult somatic cells. *MYC* activation alone is capable of enforcing DNA replication, but not cell division (Johnston et al. 1999; Felsher et al. 2000). Mitotic arrest may represent a critical fail-safe mechanism. If *MYC* were capable of enforcing mitotic division as well as cell growth and DNA replication, then aberrant activation of *MYC* alone would be sufficient to induce tumorigenesis. Activation of *MYC* in immature hepatocytes, cells already committed to a program of cellular proliferation, is sufficient to induce tumorigenesis. Activation of *MYC* in adult cells must require additional genetic events that permit mitotic cell division. Notably, our interpretation of our findings is supported by similar observations previously described in keratinocytes (Gandarillas et al. 2000).

We now can offer an explanation for what previously have been described as discordant results between reports that the *MYC* oncogene regulates cellular proliferation and other reports that *MYC* regulates cell growth (Mateyak et al. 1997; Iritani and Eisenman 1999; Johnston et al. 1999; Grandori et al. 2000; Kim et al. 2000; Trumpp et al. 2001). *MYC* generally appears to coordinate cell growth with DNA replication (Mateyak et al. 1997; Iritani and Eisenman 1999; Johnston et al. 1999; Grandori et al. 2000; Kim et al. 2000; Trumpp et al. 2001). However, contrary to what has been described, we show that the ability of *MYC* to induce cell division may depend on the developmental context of the cell. In adult hepatocytes, which normally do not proliferate, *MYC* activation can induce cell growth and DNA replication, but not cell division. In embryonic or neonatal hepatocytes, which are intrinsically committed to cellular proliferation, *MYC* activation induces cell growth, DNA replication, and mitotic division. Hence, the consequences of *MYC* activation appear to depend on the previous commitment of cells in a specific developmental state to a cellular program capable only of growth versus growth and cellular proliferation. It is not clear whether the hepatocytes that ultimately give rise to the tumors we observed derive from mitotically arrested diploid hepatocytes or from polyploid hepatocytes that acquire the capacity to undergo mitotic division.

### MYC Is Restrained from Inducing Proliferation by an Arrest in Cell Division

Many reports document that p53 functions as a general surveillance checkpoint that prevents oncogenes from inducing tumorigenesis (Sherr 1998; Vogelstein et al. 2000; Wahl and Carr 2001). *MYC* activation is known to induce *p53* function (Chernova et al. 1998; Zindy et al. 1998; Schmitt et al. 1999; Felsher et al. 2000; Grandori et al. 2000; Oster et al. 2002; Pelengaris et al. 2002a, 2002b), which in turn has been shown to cause apoptosis (Evan et al. 1992; Zindy et al. 1998; Schmitt et al. 1999; Grandori et al. 2000; Oster et al. 2002; Pelengaris et al. 2002a, 2002b). In contrast to these reports, we found that *MYC* is restrained from causing cell division in adult hepatocytes, at least in part, through a p53-dependent mechanism. Our results are consistent with many reports that demonstrate that p53 regulates checkpoints during DNA replication and during mitosis (Wahl and Carr 2001).

We conclude that apoptosis is not the mechanism that precludes *MYC* from inducing neoplasia in hepatocytes. Rather, apoptosis is associated with *MYC*-induced neoplastic progression. Notably, similar results have been described in MYC-induced breast cancer and lymphoma (McCormack et al. 1998; Blyth et al. 2000). There are several possible explanations for this discordance between our results and many previous reports. *MYC* may only induce apoptosis in some cellular lineages or only in particular differentiative contexts. In this regard, we have shown that upon inactivating *MYC*, tumor cells differentiated, and upon *MYC* reactivation in this new differentiative state, cells underwent apoptosis (Jain et al. 2002). Thus, whether oncogene activation induces proliferation, growth, arrest, or apoptosis may depend on the gene expression program of a cell associated with a particular differentiative state (Felsher 2003).

We found that the ability of MYC overexpression to induce tumorigenesis in adult hepatocytes often requires the loss of p53 function. *MYC*-induced HCC exhibited reduced p53 protein expression and transcriptional activity. The introduction of a single mutant *p53* allele greatly accelerated *MYC*'s ability to induce tumorigenesis. Hence, in adult hepatocytes, loss of p53 function appears to be necessary to permit *MYC* activation to induce cellular proliferation and tumorigenesis. Our results here are similar to our previous observations that normal mouse and human fibroblasts overexpressing MYC replicate and even endoreduplicate their DNA, but are unable to undergo mitotic division unless p53 function has been lost (Felsher et al. 2000).

### Specific Developmental Contexts Permit Oncogene-Induced Tumorigenesis

We may be able to explain why the frequency and spectrum of neoplasia vary with the host's age. In general, children are susceptible to tumors of the hematopoietic system, musculo-skeletal system, and central nervous system, whereas adults are susceptible more frequently to tumors derived from epithelial lineages, such as colon, lung, breast, prostate, and liver. The ability of oncogenes to induce cancer may be dependent upon the differentiative context. Frequently, oncogene activation has been associated with the malignant conversion of immature cellular compartments (Adams et al. 1985; Spanopoulou et al. 1989; Pelengaris et al. 1999; Blyth et al. 2000).

We may be able to explain why neonatal versus adult humans that have become infected with hepatitis B succumb to HCC with a 100-fold-increased frequency and reduced latency of tumor onset (Chang et al. 1991, 1997). We infer that oncogenes in general are more potent in inducing cancer in hepatocytes of younger hosts because these cells are committed to a developmental program permissive to tumorigenesis. Cancers frequently correspond to the malignant expansion of specific immature differentiative states within a given cellular lineage (Greaves 1986; Klein and Klein 1986). It would be advantageous for adult somatic cells, which are required to be long lived, to acquire mechanisms that prevent single oncogenes from inducing inappropriate cellular proliferation and thereby tumorigenesis. Two such mechanisms have been proposed: Some oncogenes induce premature senescence (Serrano et al. 1997; Lin et al. 1998; Zhu et al. 1998; Dimri et al. 2000), and other oncogenes induce apoptosis (Pelengaris et al. 2000). Here we have provided evidence for a different mechanism: The aberrant activation of proto-oncogenes, such as *MYC,* in the developmental context of adult somatic cells appears to be inherently prohibited from inducing mitotic division.

The intrinsic inhibition of a proliferative program in mature somatic cells may be a more parsimonious mechanism than apoptosis as a means of restraining tumorigenesis because it would permit otherwise normal fully differentiated somatic cells to continue to operate despite their acquisition of an activating mutation in an oncogene. In some cases, it would be advantageous to arrest cells rather than induce their apoptosis. Immature cells, which are committed to a program of proliferative expansion, are inherently more susceptible to oncogene-induced autonomous proliferation and tumorigenesis. In immature cells, apoptosis may be the only mechanism that could prevent tumorigenesis. Thus, the differentiative state and epigenetic program of a cell influences whether an oncogene induces cellular senescence, mitotic arrest, or apoptosis.

Generally, oncogenes may cause tumorigenesis most readily in developmental contexts that provide a gene expression program permissive to tumorigenesis (Jain et al. 2002; Felsher 2003). Pathologic conditions that trigger the expansion of immature cells—tissue injury and regeneration, infection, and autoimmune processes—may be associated with cancer because they change the cellular state, now permitting a single oncogene, such as *MYC,* to initiate tumorigenesis. In this regard, we found that *MYC* accelerated tumorigenesis in adult hosts that had undergone partial hepatectomy and were undergoing liver regeneration—a state that induces robust cellular proliferation. However, tumorigenesis was not accelerated to the same degree as observed when *MYC* was activated in embryonic or neonatal hosts. Therefore, inducing the capacity of a cell to proliferate alone is unlikely to be sufficient to confer susceptibility to tumorigenesis. It has long been appreciated that conferring the ability of a cell to proliferate is not sufficient to induce tumorigenesis. Other developmentally specific parameters may play a more critical role in defining when oncogene activation results in tumorigenesis. The model system we have developed should prove useful in defining how particular developmental contexts and pathologic states contribute to tumorigenesis.

## Materials and Methods

### 

#### Transgenic mice

The TRE-*MYC* transgenic line generated for these experiments was described previously (Felsher and Bishop 1999b). The LT-tTA transgenic line was kindly provided by H. Bujard (Kistner et al. 1996). The p53^+/−^ mice were generously provided by A. Bradley. Mice were mated and screened by PCR. *MYC* expression was activated by removing doxycycline treatment (100 μg/ml) from the drinking water of mice transgenic for both TRE-*MYC* and LAP-tTA.

#### Tumorigenicity assays

*MYC* was activated in the liver by removing doxycycline treatment from the water. *MYC* was activated during embryonic development by removing doxycycline treatment from the mating cage before conception. *MYC* was activated at birth by removing doxycycline from the mating cage immediately after the birth of the litter. Mice were monitored daily and were sacrificed when moribund. During necropsy, liver tissues were saved by fixation in 10% buffered formalin or by being snap frozen in liquid nitrogen. For transplantation, liver tumor specimens were sliced into small pieces, incubated first in calcium-free Hank's Buffered Saline Solution (HBSS) on a stirring plate at 37 °C for 20 min, then washed and resuspended in 1× digestion buffer with 1.5 mg/ml collagenase, and incubated on a stirring plate at 37 °C for an additional 40 min. The solution was then filtered through a 100-μm filter, washed, and resuspended in PBS twice. After the first wash, the solution was resuspended in 10 ml of PBS. After the second filtering and wash, the solution was resuspended in 500 μl of PBS. A quantity of 10^6^ cells was injected subcutaneously into *scid* mice (250 μl per injection) using a 1-ml syringe and 27-gauge needle. Mice showed signs of tumorigenesis within 2–3 weeks of inoculation. Mice were sacrificed when tumors reached 2 cm in size. To prepare 5× digestion buffer, 1.12 g of KCl, 37.94 g of NaCl, 0.69 g of NaH2PO_4_*H_2_O, and 9.9 g of dextrose monohydrate were dissolved in 1 liter of H_2_O.

#### Partial hepatectomy

The median and left lateral lobes comprise about 70% of the liver and their removal is recognized classically as a partial hepatectomy. We performed a one-third partial hepatectomy, removing only the median lobe of the liver. Mice were anesthetized with 16 mg/kg ketamine/xylazine. An incision was made through the midline ventral abdominal skin and abdominal muscles, extending from just above the xiphoid cartilage to about halfway towards the base of the tail. A small bolster was placed under the thorax, causing the liver to fall forwards away from the diaphragm. The liver was pushed out and the suspensory ligaments were cut with blunt-end scissors. The median lobe was raised and a ligature was tied around it with the blood vessels at the base. The liver lobe was removed by cutting close to the ligature with sharp scissors. The bolster was removed and the muscle and skin incisions were closed. The mice were monitored hourly for pain and dehydration after the procedure.

#### Histology

Liver tissues were fixed in 10% buffered formalin for 24 h and then transferred to 70% ethanol until embedding in paraffin. Tissue sections 4 μm thick were cut from paraffin-embedded blocks and placed on glass slides. Hematoxylin and eosin (H&E) staining was performed using standard procedures. The Stanford Histology Core laboratory prepared paraffin sections and performed H&E staining. We measured Ki67 expression by immunofluorescence using a mouse anti-human Ki67 monoclonal antibody (BD Biosciences, Palo Alto, California, United States). We used the Vector M.O.M. Basic Kit (Vector Laboratories, Burlingame, California, United States). Sections were deparaffinized with xylene and rehydrated through graded alcohol washes, followed by antigen retrieval in a microwave for 15 min in Vector Antigen Unmasking solution (H-3300). The slides were then incubated in 100 mM glycine for 2 × 8 min to reduce fluorescent background. Slides were blocked by incubation in avidin for 10 min followed by biotin for 10 min using the Dako biotin blocking system (DAKO Corporation, Carpinteria, California, United States) and subsequently incubated for 1 h in M.O.M. IgG-blocking reagent diluted 1:4 in PBS. Slides were then incubated for 1 h in mouse anti-human Ki67 monoclonal antibody diluted 1:100 in M.O.M. diluent. Slides were washed in TBST for 3 × 5 min to reduce background and were then treated with M.O.M. biotin-labeled anti-mouse IgG, diluted 1:250 in M.O.M. diluent. Following another 3 × 5 min of TBST washes, slides were incubated for exactly 45 min in Cy3-conjugated streptavidin diluted 1:800 in PBS (Amersham Biosciences, Piscataway, New Jersey, United States) in the dark. To visualize nuclei, slides were counterstained with 0.2 μg/ml DAPI. Ki67-positive cells were visualized by fluorescence microscopy.

#### Western blot analysis

Western analysis was performed using conventional techniques. Liver tissues were disrupted and protein was isolated using a pestle and tube homogenizer in NP-40 lysis buffer. Equal protein was loaded in each lane, as quantitated by the Bicinchoninic Acid (BCA) Protein Assay (Pierce, Rockford, Illinois, United States). Proteins were electrophoresed on 10% Tris-HCl polyacrylamide gels at 100 V for 60 min and transferred on PVDF membranes at 100 V for 60 min. The membrane was blocked in 5% nonfat dry milk solution in TBS at 4 °C overnight. MYC protein expression was detected using the C-19 rabbit polyclonal antibody that recognizes mouse and human MYC (Santa Cruz Biotechnology, Santa Cruz, California, United States). p53 protein expression was detected using the NCL-p53-CM5p rabbit polyclonal antibody (Vector Laboratories). As a positive control, we used a hematopoietic tumor previously shown to overexpress p53 that was generously provided by Dr. Kevin Smith.

#### Cell and nucleus size measurements

Images of H&E-stained liver sections were made with a Nikon Eclipse E800 microscope utilizing a Spot RT Slider digital camera (Diagnostic Instruments, Sterling Heights, Michigan, United States) and Spot Advanced Software (version 3.2.4, Diagnostic Instruments). To estimate cellular and nuclear volume, the radii of at least five hepatocytes per field were measured in at least three fields.

#### Hepatocyte isolation

Hepatocytes were collected by a two-step in situ perfusion technique (Seglen 1976; Bumgardner et al. 1990). First, the inferior vena cava was cannulated and the liver perfused with calcium-free EGTA buffer followed by calcium-containing collagenase buffer (Invitrogen, Carlsbad, California, United States). After the perfusion, the liver was excised and mechanically disrupted in Williams' E medium (Sigma-Aldrich, St. Louis, Missouri, United States). The resulting slurry was filtered through a 40-μm filter, and viable hepatocytes were isolated by Percoll gradient centrifugation. The pelleted hepatocytes were washed serially with Williams' E medium and counted prior to further analyses.

#### Cell size measurements by FACS analysis

Cell size was determined by FACS forward versus light scatter of isolated hepatocytes utilizing a Becton Dickinson FACSCaliber (BD Biosciences). Data were analyzed using Cellquest v3.3 Software (BD Biosciences).

#### DNA content

Nuclei were prepared for staining by touching a cut piece of liver to superfrost slides. The smears were then air dried, fixed in formalin for 5 min, and stored in 70% ethanol. The nuclei were stained for DNA content analysis according to the Feulgen technique (Oppedal et al. 1988).

#### Apoptosis assay

Apoptosis was detected using terminal deoxyribonucleotidyl transferase-mediated dUTP nick end labeling (TUNEL) staining, using the In Situ Death Detection Kit (Boehringer Mannheim, Indianapolis, Indiana, United States). In order to visualize the nuclei, cells were counterstained with DAPI (0.2 μg/ml). TUNEL-positive cells were visualized by fluorescence microscopy.

#### Northern blot analysis

Northern blotting and probing were performed using standard methods. RNA samples were isolated according to the Trizol product manual specifications using a Kontes 1-ml glass tissue homogenizer. A formaldehyde, 1% agarose gel was used to run the Northerns, and transferring was done overnight in 20× SSC. Blots were washed in 2× SSC, cross-linked twice in a Stratalinker UV source, and pre-hybed and hybridized using the UltraHyb (Ambion, Austin, Texas, United States) product specifications. cDNAs corresponding to *p21, MDM2,* and glyceraldehye-3-phosphate dehydrogenase *(GAPDH)* were used as probes (Macleod et al. 1995; el-Deiry 1998). The probes were generated through random priming reactions. Kodak BioMax MS film was used to expose the blots.

#### Loss of p53 heterozygosity analysis by PCR

To evaluate loss of heterozygosity of liver tumors derived from mice heterozygous for p53 deletion, PCR analysis was performed as previously described (Timme and Thompson 1994).

#### RNA isolation and quantification

Total cellular RNA was isolated from snap-frozen liver tissue using the Invitrogen Micro-to-Midi Total RNA Purification System according to the manufacturer's instructions. The amount of total RNA isolated from tissues was quantified using spectrophotometric OD_260_ measurements. The quality of RNA was measured on a formaldehyde, 1% agarose gel.

#### Real-time PCR

The RNA of human c-*MYC* and rodent *GAPDH* were measured by real-time quantitative RT-PCR using the 5′ nuclease technology on an ABI PRISM 7900HT Sequence Detection System (Applied Biosystems, Foster City, California, United States). The probe sequences for human *c-MYC* were forward primer 5′-CCCCTGGTGCTCCATGAG-3′ and reverse primer 5′-GCCTGCCTCTTTCCACAGA-3′. The human c-*MYC* probe, 5′-TCCTCCTCAGAGTCGC-3′, was labeled with FAM dye-MGB. A VIC TaqMan rodent *GAPDH* control reagents kit (Applied Biosystems) was used to measure mouse *GAPDH.* The RNA was reverse transcribed using the High-Capacity cDNA Archive Kit (Applied Biosystems) according to the manufacturer's protocol with a minor modification, the addition of RNase inhibitor (Applied Biosystems) at a final concentration of 1 U/μl. Samples were incubated at 25 °C for 10 min and 37 °C for 180 min. PCR reactions were prepared in a final volume of 20 μl, with final concentrations of 1× TaqMan Universal PCR Master Mix (Applied Biosystems) and cDNA derived from 20 ng of input RNA as determined by spectrophotometric OD_260_ measurements. Thermal cycling conditions comprised an initial UNG incubation at 50 °C for 2 min, AmpliTaqGold DNA polymerase activation at 95 °C for 10 min, 40 cycles of denaturation at 95 °C for 15 s, and annealing and extension at 60 °C for 1 min. Each measurement was performed in triplicate and the threshold cycle *(C_t_),* the fractional cycle number at which the amount of amplified target reached a fixed threshold, was determined. For calibration and generation of standard curves, we used cDNA prepared from Universal Human Reference RNA (Stratagene, La Jolla, California, United States) and cDNA prepared from Universal Mouse Reference RNA (Stratagene). Universal Human Reference RNA was used for human *c-MYC* and Universal Mouse Reference RNA was used for rodent *GAPDH.*

## Supporting Information

### Accession Numbers

Swiss-Prot accession numbers (http://us.expasy.org/sprot/) for the loci discussed in this paper are the following: Cdkn1a (mouse), P39689; Mdm2 (mouse), P23804; Myc (human), P01106; Myc (mouse), P01108; and Tp53 (mouse), P02340.

## Abbreviations

HCC: hepatocellular carcinoma
Tet system: tetracycline regulatory system
